# Open source libraries and frameworks for biological data visualisation: A guide for developers

**DOI:** 10.1002/pmic.201400377

**Published:** 2015-02-05

**Authors:** Rui Wang, Yasset Perez-Riverol, Henning Hermjakob, Juan Antonio Vizcaíno

**Affiliations:** European Molecular Biology Laboratory, European Bioinformatics Institute (EMBL-EBI), Wellcome Trust Genome CampusHinxton, Cambridge, UK

**Keywords:** Bioinformatics, Chart, Hierarchy, Network, Software library

## Abstract

Recent advances in high-throughput experimental techniques have led to an exponential increase in both the size and the complexity of the data sets commonly studied in biology. Data visualisation is increasingly used as the key to unlock this data, going from hypothesis generation to model evaluation and tool implementation. It is becoming more and more the heart of bioinformatics workflows, enabling scientists to reason and communicate more effectively. In parallel, there has been a corresponding trend towards the development of related software, which has triggered the maturation of different visualisation libraries and frameworks. For bioinformaticians, scientific programmers and software developers, the main challenge is to pick out the most fitting one(s) to create clear, meaningful and integrated data visualisation for their particular use cases. In this review, we introduce a collection of open source or free to use libraries and frameworks for creating data visualisation, covering the generation of a wide variety of charts and graphs. We will focus on software written in Java, JavaScript or Python. We truly believe this software offers the potential to turn tedious data into exciting visual stories.

## 1 Introduction

Data visualisation can be defined as the graphical display of abstract information for two main purposes: data analysis and communication. Data visualisation has long been an integral tool for scientific research, constituting a powerful means to discover and understand the information available in the data and to present them to others [1]. As we currently are in the ‘Big Data’ era, it becomes more important to expand our capacity to process information for analysis and communication purposes [1,2]. In a life sciences research context, good visualisation techniques can support the statistical treatment of data or even become an analysis technique, apart from being used as a communication tool. The main goal is to benefit from the natural human pattern recognition ability, and apply this through interactive software for efficient exploration and effective communication [3,4].

The development of software for data visualisation is a design process that requires multi-disciplinary skills, like engineering, statistics and graphical design. Ever since the introduction of high-throughput experimental approaches, there has been a growing appetite for the visual display of the information to help in the study of biological processes [5]. Biological researchers are constantly looking for fresh ways of drawing conclusions from complex data sets and combining them to generate new insights [6]. As a result, we are witnessing a rapid increase in data visualisation tools and software libraries developed in the context of genomics [7–11], transcriptomics [12,13], proteomics [14–16] and systems biology approaches [5,17], among other ‘omics’ fields. These tools can be broadly classified in two main categories:

- Specialised tools, where the focus is put on answering a specific or a group of related questions. For example, BioJS [18] is a recent initiative aiming to create a registry of graphical components that are easy to reuse, to represent biological information. Each component in the BioJS registry is designed to perform a particular visualisation task, such as ‘browse chromosome’ or ‘view protein 3D structure’, among many others.
- Integration tools, where the focus is to incorporate multiple data types, organize and integrate them under one single framework. An exemplary case is Cytocape [19,20], an open source software platform for integrating bimolecular interaction networks. It allows experimentalists to overlay annotations, gene expression profiles and other data types into these networks, enabling them to perform large-scale analysis without much informatics and programming expertise.

In parallel to biology, the field of data visualisation is constantly evolving and considerable progress has been made in integrating and displaying highly complex data [2,6]. Coupled with the development of high-performance web browsers and interactive visualisation [21] using JavaScript and HTML5 [22], a diverse range of high-quality visualisation tools and libraries are being created, developed and continuously enhanced. However, despite the vast improvement in biological data visualisation tools and libraries, two major challenges remain at present:

- How to benefit from this multi-scale complex data without being overwhelmed [23]. Clear objectives are needed to drive the design process in order to truly benefit from the visualisation.
- The advances in visualisation are not adequately described and shared within the biological scientific community [24,25]. Without the help of the visualisation practitioners, it can be a daunting task for scientists to determine the best visualisation option among the vast range of choices.

In fact, due to the lack of understanding of what is available and the lack of standardisation in biological data visualisation, scientists often decide to develop their own customised solutions. However, as regularly happens in software development, this is often the most costly route [26]. To achieve meaningful and efficient data visualisation requires substantial investment in, e.g. biological expertise, user feedback and development time. In most cases, this is beyond the capacity of most research groups. Beyond the obvious need to try and practice, we first need to know and understand well the tools and libraries available [26].

Taking this into account, in this review we will try to bridge the existing gap between the ever-increasing requirements for biological data visualisation and the overwhelming range of open source or free to use visualisation libraries and frameworks currently available. We will focus our efforts in three key areas: (i) Charts, the most frequently used visualisation techniques; (ii) Networks, widely used in systems biology and ‘omics’ data integration and (iii) Hierarchies, which employ tree structures for visualising hierarchical data. We will cover software written in Java, JavaScript or Python. Of course, there is software written in other programming languages. For instance, the R programming language (http://www.r-project.org/) related software is explained in detail in another manuscript in this special issue.

Another important consideration is that the field of data visualisation is evolving very rapidly. It is important to highlight that this manuscript is aimed at bioinformaticians, scientific programmers and/or software developers. As such, we want to make the reader aware that a certain technical background is needed to follow and fully understand the text. We hope this manuscript will help them to find the right software for their purposes. The source code and the corresponding data to all the original figures generated are available at https://github.com/ypriverol/visualisation-manuscript-examples.

## 2 Data visualisation in life sciences

The design of visualisation techniques is a process guided by three different stages: (i) determine which questions to ask; (ii) identify the appropriate relevant data and (iii) select effective visual encodings to map data values to graphical features (e.g. position, size, shape and colour). One of the main challenges is that, for any given data set, the number of visual encodings (and thus the space of possible visualisation designs) is extremely large. To guide this process, computer scientists, psychologists and statisticians have studied how well different data encodings facilitate the comprehension of different data types such as numbers, categories and networks. This process has led to the emergence of new mathematical models and algorithms such as principal component analysis, clustering, self-organized maps or hierarchical aggregation algorithms. At the same time, selecting the right set of visual components must be done in combination with the data compression and encoding processed. For instance, to represent a tree structure, the data must be encoded in hierarchical data structures using different algorithms like for instance, k-means, fuzzy or k-nearest neighbours. For that reason, the evolution of both fields: data visualisation and computer algorithms has gone in parallel in recent years [2,23].

**Figure 1:**
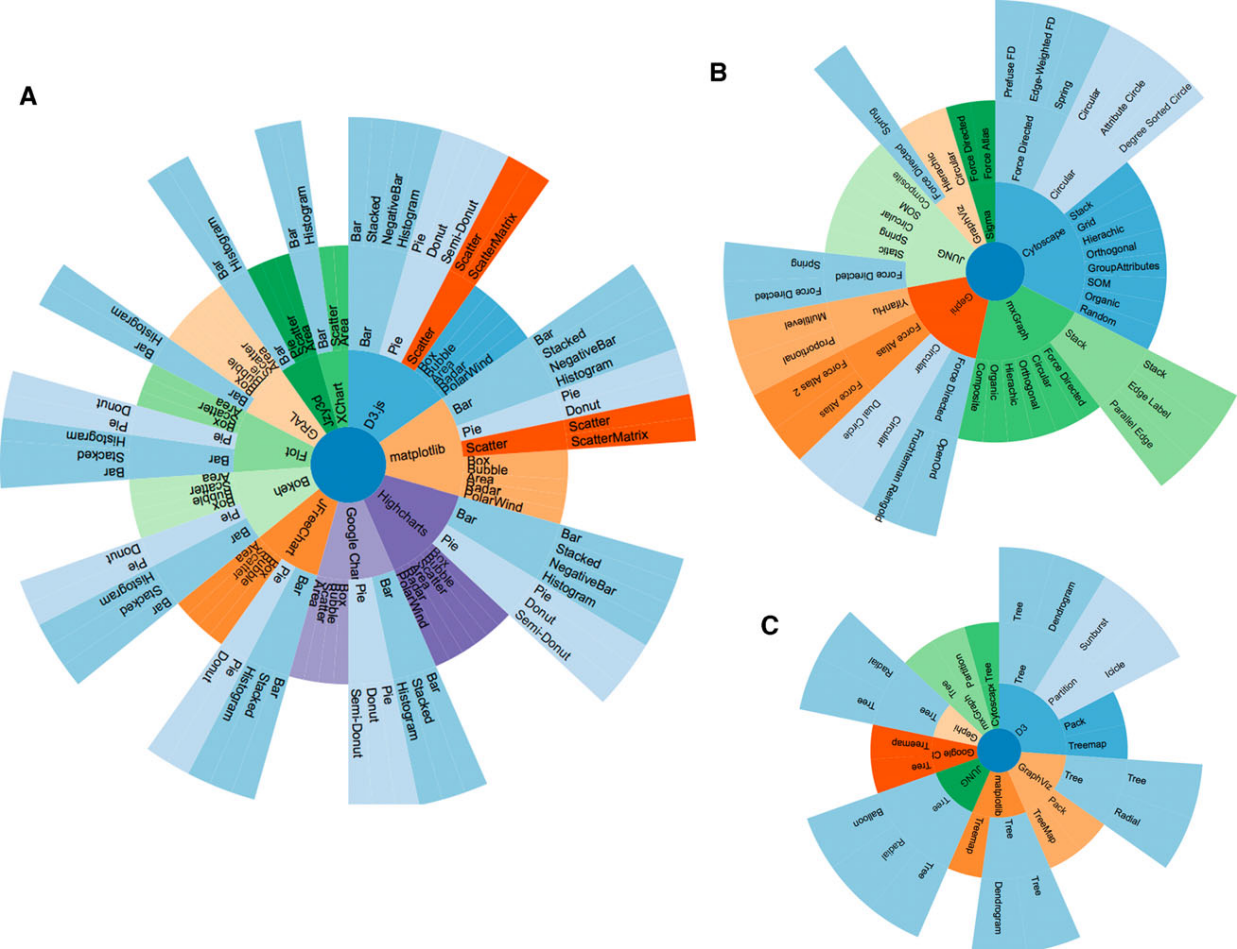
General comparison of features among the different libraries described in the main text: (A) charts, (B) networks, and (C) hierarchical representations. The figure was generated using the *D3.js* library.

Different taxonomies or classifications of data visualisation methods and techniques have been proposed [27–29]. Figure1 shows different data visualisation techniques, the software libraries that implement and develop those concepts, and the charts and plots used in the implementation. The seven categories proposed in the Shneiderman classification [29] were condensed to three main categories in our classification:

- Charts: Multidimensional charts and plots are data in which items with *n* attributes become points in an *n*-dimensional space.
- Networks: Structures where items are linked to an arbitrary number of other items.
- Hierarchies: Collections of items in which each item has a link to one parent or child item.

This conceptual chart (Fig.1) will guide the readers throughout the contents of this review.

## 3 Charts

Multi-dimensional [29] visualisation methods can be used to represent and plot data. Among the possible choices, charts comprise probably the most popular techniques, since they are very effective in presenting 2- or 3D data. There are many types of charts: *x*–*y* (and -*z*) plots, line graphs, bar and column charts, area charts, stacked bars and column graphs, histograms, pie charts, doughnut charts, box plots and many more.

In this section, we will focus on five libraries and will also comment on several packages that support the generation of generic charts and plots for displaying multidimensional data (Table1). A list of the most common charts is described in the Supporting Information Table 1. In addition, a short description about the meaning of each chart and the corresponding software is also included there.

**Table 1:** Libraries and frameworks for data visualization split in three different categories: charts, networks and hierarchies

Name	Language	Platform	Type	Description	Supported Categories	URL
D3.js	JavaScript	Web	Open source	JavaScript visualisation framework designed to utilise the capabilities of CSS3, HTML5 and SVG	Charts/Hierarchies	http://d3js.org/
JFreeChart Orson Charts	Java	Desktop Web	Free	Java well-known library to generate charts in 2D and 3D	Charts	http://www.jfree.org/jfreechart/
Google Charts	JavaScript	Web	Free	Google project to embed many different kinds of charts and maps in web pages	Charts/Hierarchies	http://code.google.com/apis/charttools
matplotlib	Python	Desktop	Open source	Desktop plotting library written in Python for creating quality plots (primarily in 2D)	Charts/Hierarchies	http://matplotlib.org/
MPLD3	Python	Web	Open source	Python package that provides a *D3.js* based viewer for *matplotlib*	Charts	http://mpld3.github.io/
GRAL	Java	Desktop	Open source	Java library for displaying plots	Charts	http://trac.erichseifert.de/gral/
Jzy3d	Java	Desktop	Open source	Library to easily draw 3D scientific data	Charts	http://www.jzy3d.org/
XChart	Java	Desktop	Open source	Lightweight Java library for plotting data	Charts	http://www.xeiam.com/xchart
Flot	JavaScript	Web	Open source	Plotting library for jQuery	Charts	http://www.flotcharts.org/
Bokeh	Python	Web	Open source	Python interactive visualization library that targets modern web browsers	Charts	http://bokeh.pydata.org/
Cytoscape	Java	Desktop	Open source	Framework for integrating, visualising and analysing data in the context of biological networks	Networks/Hierarchies	http://www.cytoscape.org/
Cytoscape.js	JavaScript	Web	Open source	Framework for integrating, visualising and analysing data in the context of biological networks	Networks/Hierarchies	http://cytoscape.github.io/cytoscape.js
Gephi	Java	Desktop	Open source	Platform written in Java for visualising and manipulating large graphs	Networks/Hierarchies	http://gephi.github.io/
Graphviz	DOT, Java, Python, C, C++	Desktop/Web	Open source	Graph Visualization Framework for drawing graphs specified in DOT language scripts	Networks/Hierarchies	http://www.graphviz.org/
Sigma.js	JavaScript	Web	Open source	JavaScript library dedicated to graph drawing, using either the HTML canvas element or WebGL	Networks	http://sigmajs.org/
mxGraph	JavaScript	Web	Commercial	Commercial JavaScript library	Networks/Hierarchies	http://www.jgraph.com/mxgraph.html
JGraphX	Java	Desktop	Open source	Open-source Java Swing graph visualization	Networks/Hierarchies	https://github.com/jgraph/jgraphx
JUNG	Java	Desktop	Open source	A library that provides a common and extendible language for graph visualization	Networks/Hierarchies	http://jung.sourceforge.net/

### 3.1 D3.js

The open source library *D3.js* [30] (http://d3js.org/) is JavaScript-based and designed to utilise the capabilities of widely used web standards such as CSS3 (Cascading Style Sheets), HTML5 and SVG (Scalable Vector Graphics). It was created and is maintained by researchers from Stanford University's Stanford Visualisation Group. It is also known as the successor of the *Protovis* visualisation library [31]. *D3.js* targets animations, interactions and complex and dynamic visualisations trying to address the challenges derived from visualisation in the web by putting an emphasis in the efficient manipulation of HTML elements. At its kernel, it uses pre-built JavaScript functions to select elements, create SVG objects, style them or add transitions and dynamic effects to them. This approach minimizes the conceptual stack of the library and makes possible the creation of custom complex visualisations.

In addition to its kernel, *D3.js* offers four main optional modules to encapsulate reusable solutions: (i) Shapes, including a number of built-in simple shapes, such as axis-aligned rectangles and circles. These visual abstractions represent the foundations in the creation of useful charts and graphs; (ii) Scales, which contains quantitative scales for continuous input domains such as numbers, and ordinal scales for discrete input domains (for instance names or categories and time scales); (iii) Layouts, including a large selection of commonly used layouts such as the force-directed layout and (iv) Behaviours, which encapsulates common interaction techniques on use input, such as zooming and dragging.

**Figure 2:**
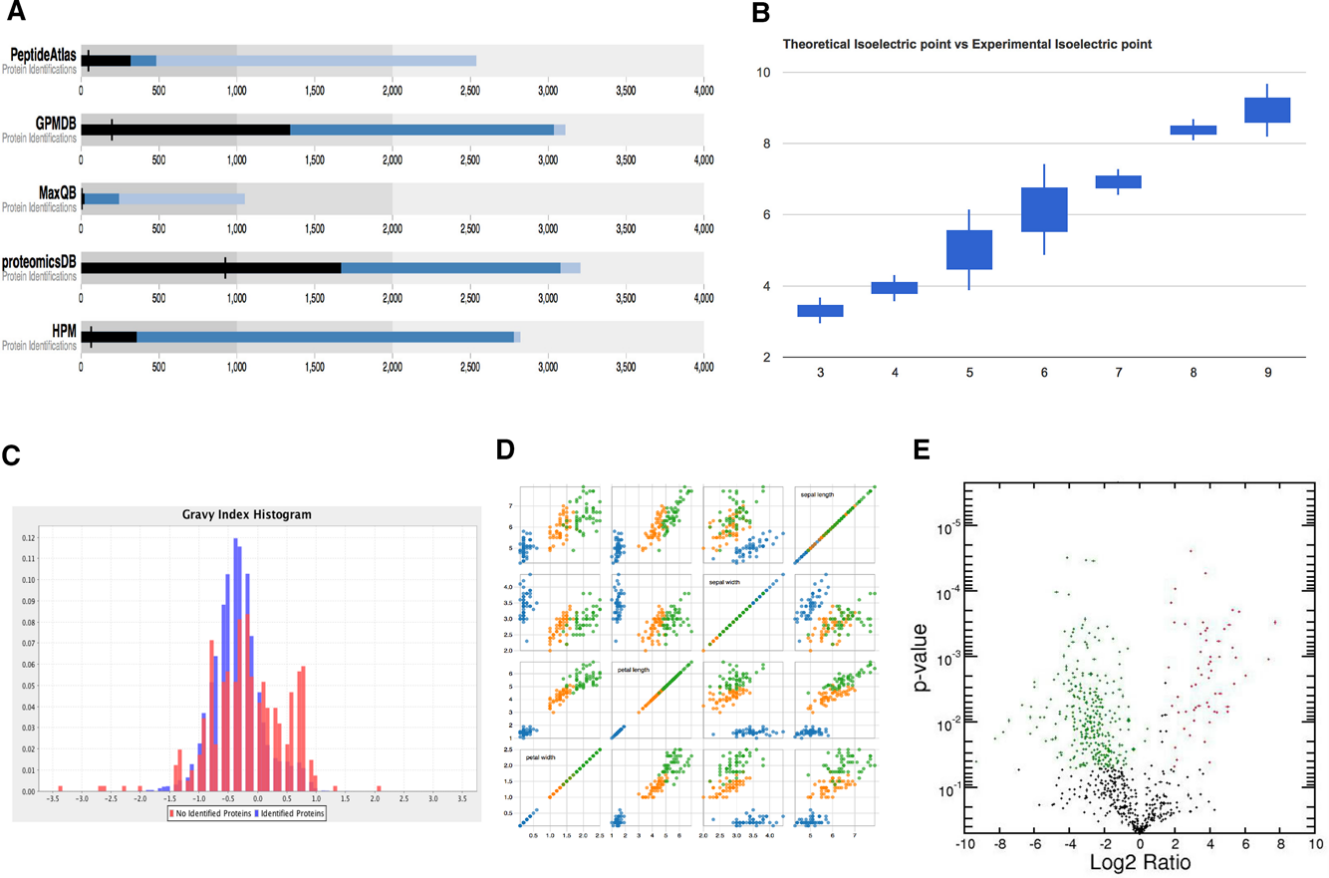
Examples of charts: (A) bullet plots of identified proteins in different MS proteomics resources (original data published in [75]); (B) box plots of theoretical isoelectric point (*x*-axis) for seven experimental fractions (original data published in [39,40]); (C) histograms of gravy index of proteins non-identified in protein expression resources (original data published in Ref. [75]); (D) scatter plot matrix charts; and (E) volcano plot. (A) was generated using *Google Charts*, and (B) was generated using *D3.js* bullet plugin (https://github.com/d3/d3-plugins/tree/master/bullet), respectively; (C) and (D) were generated using *JFreeChart* and *D3.js*, respectively; (E) was generated using *matplotlib*.

By default, *D3.js* does not generate predefined visualisations for the end users. Its functionality can be extended using many available plugins (https://github.com/d3/d3-plugins). Many of them are aimed at creating charts: for instance, the ‘box plugin’ (for creating box plots, https://github.com/d3/d3-plugins/tree/master/box) and the ‘bullet plugin’ (for bullet charts, Fig.2A, https://github.com/d3/d3-plugins/tree/master/bullet).

In addition to these extensions, there are a number of libraries for charting that have been built using *D3.js*. Among them (i) *Dimple* (http://dimplejs.org/) provides an object-oriented API (Application Programming Interface) for business analytics; (ii) *NVD3* (http://nvd3.org/), a project attempting to build re-usable charts and chart components and (iii) *Crossfilter* (http://square.github.io/crossfilter/), a library for exploring large multivariate data sets in web browsers.

In the context of biological data visualisation, *D3.js* has already been used for the creation of charts in several bioinformatics projects. Recent examples include: (i) *PIQMIe* [32] (http://piqmie.semiqprot-emc.cloudlet.sara.nl/), a web server for performing semi-quantitative proteomics data management and analysis; (ii) *Genome3D* [33], a UK collaborative project created to annotate genomic sequences with predicted 3D structures based on the SCOP (Structural Classification of Proteins) and CATH (Class, Architecture, Topology, Homology) classifications; (iii) the BioJS [18] components ‘DNAContentViewer’ (http://www.ebi.ac.uk/Tools/biojs/registry/Biojs.DNAContentViewer.html) and ‘wigExplorer’ (http://www.ebi.ac.uk/Tools/biojs/registry/Biojs.wigExplorer.html) and (iv) the TGAC Browser (http://www.tgac.ac.uk/tgac-browser/), a new web-based genome browser with novel rendering and annotation capabilities. It uses *D3.js* and the *jQuery* library (http://jquery.com/).

### 3.2 JFreeChart

*JFreeChart* [34] (http://www.jfree.org/jfreechart/) is a free to use (not open-source) Java library that enables developers to easily generate graphs and charts. David Gilbert started the project in 2000 and it is arguably the most widely used chart library in Java. Many products use it such as *Paralog* (http://www.paralog.net/) and *qStudio* (http://www.timestored.com/qstudio/). The library can be used to generate the most common chart types including pies (2D and 3D), bars, bubbles, scatters (2D and 3D), histograms (Fig.2C) and Gantt charts. In addition, the API supports many interactive drawing features such as tool tips, colour gradients, drop-shadows and zooming.

*JFreeChart* provides an excellent choice for Java Swing-based applications. The main data structure in the library is the *Data set* object, which contains the data to be displayed in the charts. *JFreeChart* features many different *Data set* objects, which implement the *Data set* interface and can be used to create different types of charts such as *XYBarDataset* (Bar plot) or *DefaultPieDataset* (Pie chart). The chart can be customised on attributes like zooming, labels, colours or tool tips. The main strengths of *JFreeChart* are the extensive documentation, example code available, few dependencies and flexible customisability. Furthermore, it is capable of exporting to various common formats such as JPEG, PNG and PDF, and it can be used in JSP (Java Server Pages)/servlet-based applications to dynamically stream charts to web pages. Recently, the *JFreeChart* development team created the *Orson Charts* (http://www.object-refinery.com/orsoncharts/), a 3D chart library in Java that can generate a wide variety of 3D charts in client-side (Java FX and Java Swing) and server-side applications.

In bioinformatics projects, a number of tools have been developed using *JFreeChart* [35,36]. For instance, *SimBoolNet* draws the changes of network nodes in a time series chart, and a scatter plot [35]. *JProGO* is a tool for functional interpretation of prokaryotic microarray data that uses *JFreeChart* to draw the expression profile of the genes belonging to a specific GO node. In proteomics, the PRIDE Inspector [14] tool was designed for data analysis and visualisation of MS results. PRIDE Inspector provides a set of charts than can be used to evaluate some aspects related to the experimental data. Similar to PRIDE Inspector, different tools such as Rover [37], FragmentationAnalyzer [38] and PeptideShaker (https://peptide-shaker.googlecode.com/) have been developed to analyze MS proteomics results and are also using the *JFreeChart* library.

### 3.3 Google charts

*Google Charts* (http://code.google.com/apis/charttools/) is a free to use (not open source) JavaScript-based visualisation tool developed by Google. It allows both non-expert users and developers to embed many different kinds of charts and maps in web pages. Going from simple line charts to complex hierarchical tree maps, the selection includes a large number of ready-to-use chart types. The most common way to use *Google Charts* is by placing JavaScript snippets embedded in web pages. The charts can read data directly from a web page, retrieved from a backend database or stored in Google Spreadsheets. Charts are rendered using HTML5/SVG technology to provide cross-browser compatibility including VML (Vector Markup Language) for older Internet Explorer® versions and cross-platform portability to iOS and Android devices. Supporting Information Table 1 contains a list of the charts supported by *Google Charts* in the July 2014 version.

*Google Charts* requires data to be uploaded in a JavaScript class called *DataTable*, a 2D table containing rows and columns where each column has a data type. Developers should organize the *DataTable* in a format required by the chart (for instance both the bar and pie charts require a two-column table where each row represents a slice or bar). Developers can also query a web site that supports the ‘Chart Tools Data Source Protocol’, e.g. a Google Spreadsheet page (https://developers.google.com/chart/interactive/docs/queries). One of the key features of *Google Charts* is that all the chart types can be populated with data using the *DataTable* class, making it easy to switch between chart types as the developer experiments to find the ideal chart. It also provides methods for sorting, modifying and filtering data. Every chart has many customisable options including title, colours and different options for lines and background fills. Figure2B shows a box plot generated by *Google Charts* containing the theoretical isoelectric points of seven fractions of an off-gel electrophoresis [39,40].

*Google Charts* has been used in genomics and proteomics related tools as a platform to generate plots and charts [41–44]. For example, *EpiExplorer* [41] (http://epiexplorer.mpi-inf.mpg.de/) is web application for exploring genome and epigenome data that uses *Google Charts* for interactive data visualisation. *CircaDB* (http://circadb.org) [44] is a well-structured database of circadian transcriptional profiles from time-course mouse and human expression experiments, where data visualisation is accomplished by using pre-formatted URI (Uniform Resource Identifier) requests to *Google Charts*. Another example is the EMBL-EBI (European Bioinformatics Institute) Metagenomics portal (http://www.ebi.ac.uk/metagenomics/) [42] which allows biologists to submit raw nucleotide reads for functional and taxonomic analysis by a state-of-the-art pipeline, and store them in the European Nucleotide Archive (ENA). The ‘taxonomy analysis’ tab in this resource uses *Google Charts* to display information in a simple pie, bar or a stacked bar chart.

### 3.4 matplotlib

The open source library *matplotlib* [45] (http://matplotlib.org/) is written in Python and is aimed at creating publication quality plots (primarily in 2D). This project was started by John Hunting in 2002, originally as a package to emulate the MATLAB [46] (http://www.mathworks.co.uk/products/matlab/) graphics commands. The idea was to address the limitations involved in writing complex interactive applications in MATLAB by taking advantage of Python as the programming language. Its most important feature is the ability to produce quality 2D plots easily. In addition, these plots are embeddable in a graphical user interface (GUI) for application development, and its code is generally easy to understand and to extend. Another key aspect of *matplotlib* is its ability to support many operating systems and different graphics data backends. This has led to a large user community and to a powerful collection of scientific tools.

*matplotlib* consists of three parts: (i) the *pylab* interface, a collection of functions that allow users to create plots using code which is quite similar to the MATLAB figure generating code syntax; (ii) the *frontend*, a collection of classes that performs the actual plot creation, including managing figures, text, lines, etc. and (iii) the *backend*, which provides device-dependent handles that transform the front-end representation to a device specific output. For instance, it supports the creation of SVG graphics, PDF documents or PNG images.

*matplotlib* is often regarded as Python's equivalent of *ggplot* [47] for the R programing language. It performs well in creating various types of static images due to its cross-platform and multi-backend design approach. In addition to writing Python scripts, developers can use *matplotlib* in an interactive Python shell such as *ipython* (http://ipython.org/) to dynamically update the plots.

The package *MPLD3* (http://mpld3.github.io/), also written in Python, provides a *D3.js* based viewer for *matplotlib*. The main motivation behind *MPLD3* is to bring interactive *matplotlib* graphics to the web browser by exporting *matplotlib* to HTML code. It contains a Python module and a stand-alone JavaScript library built on *D3.js*, known as *mpld3.js*. The Python module contains a set of routines that can parse the *matplotlib* plot and output it into JSON (JavaScript Object Notation) descriptions. This JSON representation of the plots can then be parsed by *mpld3.js* and visualised in a web browser. Many of the core features of *matplotlib* are supported by *MPLD3* and additional interactivity for the plots can be extended using *MPLD3*'s own plugin framework. It also provides several plugins by default, such as the reset, zoom and the box zoom ones.

*matplotlib* found early institutional support from astronomers at the Space Telescope Science Institute (http://www.stsci.edu/portal/) and NASA's Jet Propulsion Laboratory (http://www.jpl.nasa.gov/). It is now also widely used for plotting in a number of bioinformatics projects. For instance, *Biopython* [48] (http://biopython.org/) provides a set of freely available tools for biological computation and *matplotlib* can be executed as part of the library.  *matplotlib* has also been used in *CING* [49], which is an integrated residue-based structure validation program suite. Recently, *matplotlib* has been successfully used [50] to represent the demographic history of multiple populations using multidimensional SNP (Single Nucleotide Polymorphism) frequency data sets.

### 3.5 Other libraries

Many other libraries are also available for generating charts. For instance, written in Java, *GRAL* (GRAphing Library, http://trac.erichseifert.de/gral/) is a free to use and lightweight library for displaying plots (graphs, diagrams and charts), implementing most of the charts included in *JFreeChart*. Other example is the Java library *Jzy3d* (http://www.jzy3d.org/), which allows drawing scientific data in 3D including surfaces, scatter plots, bar charts and a lot of other 3D primitives. The API provides support for rich interactive charts with colour bars, tooltips and overlays. Relying on *JOGL2* (http://jogamp.org/jogl/), developers can easily deploy native OpenGL (http://www.opengl.org/) charts on Windows, Unix, Mac OS and integrate them into Java Swing, AWT (Abstract Window Toolkit) or SWT (Standard Widget Toolkit). This library has been used as a 3D visualisation package with interactive peak exploration for chromatograms in *Maltcms* [51,52], a modular application toolkit for chromatography, MS and metabolomics.

*XChart* (http://www.xeiam.com/xchart) is another lightweight Java library for plotting data. This library is part of the ‘AHaH’ project [53], a new approach to computing where memory and processing are combined. The *XChart* library focuses on simplicity and ease-of-use, requiring only two lines of code to save or display a basic default chart.

*Flot* (http://www.flotcharts.org/) is a pure JavaScript plotting library based on the *jQuery* framework, with a focus on simple usage, attractive looks and interactive features. It works in all web browsers that support HTML5 canvas (which means most of the popular ones). *Flot* is supporting chart generation in a number of tools such as WholeCellViz [54], which can perform data visualisation for whole-cell models, and HapMap-CN [55] a CouchDB-based database which allows automated gene-centric annotations, and provides a web interface to query copy number variation from three HapMap data sets.

*Bokeh* (http://bokeh.pydata.org/) is a Python interactive visualisation library that targets modern web browsers. Its main goal is to provide concise construction of novel graphics based on the ideas of the *Grammar of Graphics* [56], *Protovis* and *D3.js*. It can also deliver this capability with a high-performance interactivity over very large or streamed data sets. This functionality is implemented via a declarative data transformation scheme and is engineered to operate in a client/server model for the modern web. Recently, *Biopython* [48] has started to develop visualisation packages using *Bokeh*.

### 3.6 Remarks

With the emergence of multivariable data sets, data scientists are trying to obtain an integrated understanding of data distributions and investigate the inter-relationships between different data attributes [57,58]. For instance, *matrix* (Fig.2D) [59,60] is an extension of a scatter plot for multidimensional data where a collection of scatter plots is organized in a matrix to provide correlation information among the attributes. This is particularly effective in pinpointing specific variables that might have similar correlations to particular genomics [61], proteomics [62] or metabolomics [63] data. Another type of scatter plot is the volcano plot [64] (Fig.2E), which is commonly used to quickly identify changes in large data sets composed of replicated data. These plots are increasingly used in analysing ‘omics’ experiments where many thousands of replicated data points are represented [65,66]. Volcano plots are constructed by plotting the negative log of the *p*-value in the *y*-axis versus the log of the fold change between two conditions. This results in data points with low *p*-values (highly significant) appearing towards the top of the plot. The log of the fold-change is used so that changes in both directions (up and down) appear equidistant from the centre [64]. Also, ‘parallel coordinates’ charts [67,68] are a common way of visualising high-dimensional geometry and analysing multivariate data. The main idea is to show a set of points in an *n*-dimensional space as *n* parallel lines, typically vertical and equally spaced. One point in an *n*-dimensional space is represented as a polyline with vertices on the parallel axes. The position of the vertex on the *i*th axis corresponds to the *i*th coordinate of the point. This visualisation is closely related to a time series, except in that it is applied to data where the axes do not correspond to points in time, and therefore do not have a natural order [68] (Supporting Information Fig. 1).

## 4 Networks

Node-link diagrams represent a powerful way of understanding entity relationships by showing their overall structure, the topology of the network [69,70]. It is a natural human trait to spot visual similarity and proximity as meaningful. Node-link diagrams have the advantage of preserving the local detail of the relationships. They make easier to identify the nearest neighbours for a particular node and look for the shortest path between two nodes. In biology, it is common to represent molecules or other biological entities as nodes and their interactions or processes as edges. Typical examples are protein–protein interaction networks and pathways.

There is already a diverse set of tools for interpreting biological networks and for curating pathways [71,72]. These have been already described in previous reviews [71,73,74] and are out of the scope in this manuscript. Instead, we target primarily six different libraries/frameworks and discuss several additional packages that support the generation of networks (Table1). A list of the most common network layouts together with a short description is included in the Supporting Information Table 2.

### 4.1 Cytoscape

**Figure 3:**
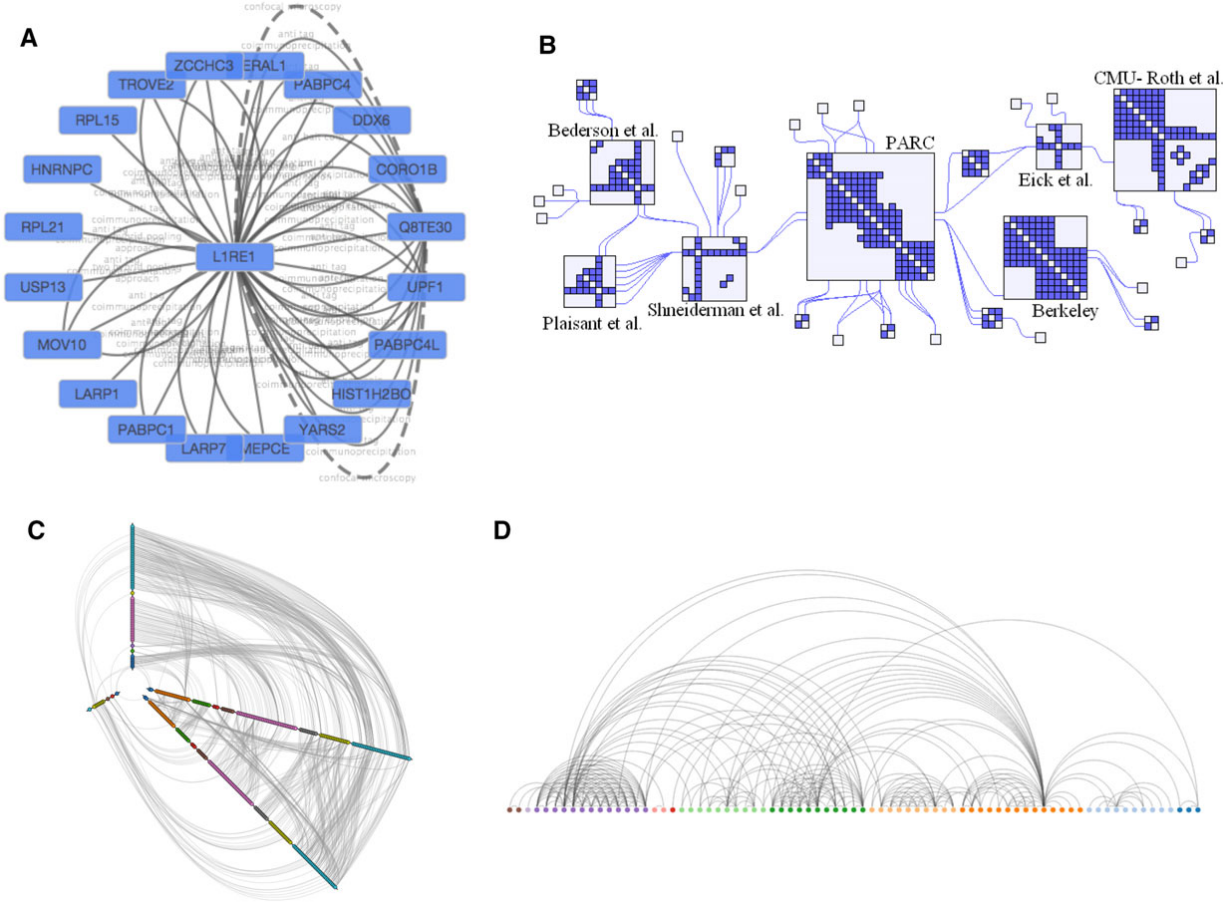
Examples of networks: (A) Network circle layout of a protein network generated with *Cytoscape;* (B) Hybrid network, generated using *NodeTrix* (http://www.aviz.fr/Research/Nodetrix), combining a network with an adjacency matrix; (C) Hive plot (http://bost.ocks.org/mike/hive/), generated using *D3.js;* and (D) Arc diagram (http://bl.ocks.org/sjengle/5431779), generated also using *D3.js*.

*Cytoscape* [17] (http://www.cytoscape.org/) is an open source desktop tool for integrating, visualising and analysing data in the context of biological networks. Since its initial release in 2002, it has grown into the de facto tool for visualising molecular interaction networks and biological pathways. By July 2014, the release of *Cytoscape* is the 3.x series. Designed from ground up using modular software architecture for long-term maintainability, the new release is aiming to replace the popular 2.x series. The 3.x series is capable of navigating large networks with more than 100 000 nodes and edges, and can layout networks in two dimensions using a variety of layout algorithms including circular, force-directed and stack layouts. Figure3A shows an example of a network graph using a circle layout containing proteins (data taken from [[75]]).

*Cytoscape* is also a software platform that has attracted a large vibrant community of active contributors. It consists of two key programming components: the *Cytoscape App API* and *cytoscape.js*. The *Cytoscape App API* (https://github.com/cytoscape) provides a programmatic way of extending and customising the *Cytoscape* desktop tool. Through the API, developers can access directly to its core data models (which represent networks and tables). Additionally, they can extend its view model to customise the network visualisation and layout. Moreover, it is possible to take advantage of *Cytoscape*'s core application framework to provide a better interoperability within the whole tool.

A large number of Apps have been developed and made available by the community (http://apps.cytoscape.org/). Several of them are well known in the analysis of biological networks [17]. For example, BiNGO [76] is frequently used for GO annotation and related ontology enrichment analyses. It can calculate overrepresented functions in the network and display them as GO directed acyclic graphs. Another example is MCODE [77], used for clustering and graph analysis. It is capable of locating clusters in highly interconnected regions of a network on the basis of vertex weighting.

*Cytoscape.js* (https://github.com/cytoscape/cytoscape.js) is an open source graph library, written in JavaScript as a jQuery plugin. It is the successor of *Cytoscape Web* (http://cytoscapeweb.cytoscape.org/), which is based on Adobe® Flash. At the moment of writing, the developer team is working actively to port all the features of *Cytoscape Web* into *Cytoscape.js*.

*Cytoscape.js* shares many design principles of the *Cytoscape App API*. For instance, it separates the graph style from data, provides core functionality and allows extensions adding functionality on top of the library. *Cytoscape.js* enables developers to display and manipulate rich, interactive graphs. It comprises many useful features including the main gestures, such as pinch-to-zoom, box selection and panning, and support for different graph theory user cases, including among others directed graphs, undirected graphs, mixed graphs, multi graphs and compound graphs. *Cytoscape.js* is intended for programmers, it is therefore not an App for end-users. Software development is necessary to integrate it into graph centric web applications. The *Cytoscape.js* API uses many JavaScript language idioms and is event-based. Furthermore, JSON definitions of elements can be used and also, nodes can be chosen with selectors that are modelled on CSS selectors and the jQuery API. With the latest release of the *Cytoscape* desktop, network data can be exported in JSON format and visualised in a web page.

Unlike *Cytoscape* Web, which is currently used in many bioinformatics projects (e.g. GeneMANIA [78], iRefWeb [79] and Pathguide [80]), at present *Cytoscape.js* has not been widely adopted yet due to its recent development. However, it is rapidly gaining momentum and expected to be widely adopted, like its predecessor [81,82].

### 4.2 Gephi

Popular for data journalism and many scientific domains, *Gephi* [83] (http://gephi.github.io/) is an open source Java based platform for visualising and manipulating large graphs. Originally created at the University of Technology of Compiegne (France), it is now maintained by the *Gephi* Consortium, which also supports the development of future releases. *Gephi* is often regarded as the ‘Photoshop®’ for networks and graphs. Users can interact with the graph representation and manipulate its structure, shape and colour. Network statistics can also be calculated (such as average degree and modularity) and the results can be overlaid on the original graph. *Gephi* has a built-in OpenGL engine. Therefore, it is able to visualise very large networks of up to a million elements and all the actions (such as layout, filtering and clustering) can happen in real-time. Additionally, *Gephi*'s software architecture is built on top of the ‘NetBeans’ platform (http://netbeans.org/). This makes that it can be extended or reused easily through well-written APIs.

One of the major strengths of *Gephi* is the availability of many plugins, which can extend and complement the core library (https://marketplace.gephi.org/). For instance, at the moment of writing *Gephi* offers 19 additional layouts via plugins. Some of them are *OpenOrd*, *Circular*, *Layered*, *Force Atlas 3D*, *Network splitter 3D* and the *Isometric* layouts. In addition, the exporter plugins are worth highlighting. They provide a way of bringing *Gephi* components from a desktop application into a web environment. For instance, the *Sigma.js* plugin can export the network from *Gephi* to a predefined *Sigma.js* library template. Developers can also choose among many options including search, group selection or explanatory text. The entire process is straightforward, as developers do not have to write any HTML/JavaScript code and only need to export the graphs from *Gephi* as HTML files.

Compared to *Cytoscape*, *Gephi* is a general network and graph visualisation tool. This makes it more useful in other disciplines outside biology. For instance, it has already been used in research projects such as DyCoNet [84], a *Gephi* plugin for identifying the modular organisation of dynamic complex networks. Another example is ForceAtlas2 [85], a continuous graph layout algorithm for network visualisation.

### 4.3 Graphviz

*Graphviz* [86] (http://www.graphviz.org/) is an open source software package initiated by AT&T Labs Research (http://www.research.att.com/) for visualising connectivity graphs. It provides graph visualisation for tools and websites in different domains like software engineering [87], networking [88], knowledge representation [89,90] and bioinformatics [91]. The core of *GraphViz* contains the implementation of a collection of common types of graph layouts, which can be used via a programming interface, command line tools, GUIs and web browsers. Very importantly, *GraphViz* also has a graph description language called *DOT* (Graph Description Language, http://www.graphviz.org/content/dot-language) and a set of tools that can generate and process *DOT* files.

In addition, *DOT* is a scriptable, batch-oriented graphing tool. Developers can write descriptions of structured information in *DOT* and the *GraphViz* layout engine can generate the output graphs in different formats. Some example formats are SVG (for web pages), PDF or Postscript (for inclusion in other documents or for display purposes in an interactive graph browser) [92]. One of key advantages of using *Graphviz* is that it can efficiently render large graphs. In fact, graphs of moderate to very large size (up to 70 000 nodes and half a million edges) can be drawn using a scalable multilevel force directed algorithm called *sfdp*.

Numerous tools and libraries have been created that can complement *Graphviz*, including different graph generators, post processors and interactive viewers. There are also high-level systems and websites that rely on *Graphviz* as a visualisation service. For instance, it has modules for content management systems like Drupal and WordPress. It also provides a large number of language bindings for Python, JavaScript, Java, Ruby, Perl and C#, where programmers can take advantage of the *Graphviz* features using their favourite programming language.

*Graphviz* has already been used in several bioinformatics projects, such as PubGene [93] and the Protein Interaction Extraction System (PIES) [94]. Another example is RGraphviz [95] (http://bioconductor.wustl.edu/bioc/html/Rgraphviz.html), included in BioConductor, which also incorporates *Graphviz* as a rendering module, along with other graph libraries.

### 4.4 Sigma.js

The open source JavaScript library *Sigma.js* (http://sigmajs.org/) is backed by the company ‘Sciences-Po Medialba’ (http://www.medialab.sciences-po.fr/). The library is dedicated to graph drawing, using either HTML5 canvas or WebGL (Web Graphics Library, http://www.khronos.org/webgl/). It has been specially designed to display interactively graphs exported from external software (like *Gephi*) and also to display dynamically graphs that are generated in real time. *Sigma.js* is lightweight and does not require any external dependencies. In fact, it is relatively easy to use and integrate into existing web applications. The default configuration of *Sigma.js* provides a variety of build-in features, like HTML5 canvas and WebGL renderers, mouse and touch support. At its core, *Sigma.js* is a rendering engine and it does not include many graph theory related features ‘out-of-the-box’, such as layout and clustering.

The *Sigma.js* API makes it possible to modify and enhance the data, refresh the rendering and listen to events. For more complex interactions, like in the case of *Cytoscape* and *Gephi*, it is possible to develop and use plugins that can add features to the core library. For instance, there are plugins to perform force-directed layout and enable the dragging of the nodes. *Sigma.js* is also scalable, taking advantage of HTML5 canvas functionality by using frame injection. For displaying large graphs on the web, *Sigma.js* does not suffer from freezing issues as other graph libraries often do. However, developers should be aware that it might still take some time to draw an entire large graph.

In our opinion, *Sigma.js* is best suited for projects where it is only required to display networks containing some basic interactions. Different bioinformatics tools have already used this library. One example is NetworkAnalyst [96], a tool for protein–protein interaction network analysis and visual exploration. Another one is NeXO Web [97], the NeXO ontology database and related visualisation platform.

### 4.5 mxGraph

Formally known as *jGraph*, *mxGraph* (http://www.jgraph.com/mxgraph.html) constitutes a family of libraries that provides features for displaying interactive diagrams and graphs. *mxGraph* is not designed specifically to be ready to use in applications. Instead it provides a range of commonly required functionalities to draw, interact with and associate a context with a diagram. Visualisation is one of main strengths of *mxGraph*, since it can represent nodes in shapes, images, vector drawing and animations. Developers can then interact with the *mxGraph* generated diagrams through a series of actions like for instance dragging and cloning cells, resizing and reshaping, connecting and disconnecting, drag and dropping, or in-place editing. In addition *mxGraph* offers a flexible API for developers to program the behaviour of these interactions. The library provides a basic set of implementations of graph layout algorithms including trees, force-directed and hierarchical layouts. However, graph analysis techniques such as clustering, decomposition and optimisation have not yet been implemented in the core *mxGraph* libraries.

By July 2014, *mxGraph* is released in two different packages: a commercial JavaScript library and an open source Java Swing visualisation library called *JGraphX* (https://github.com/jgraph/jgraphx).

The JavaScript commercial version of *mxGraph* includes one JavaScript file that contains all the functionality provided by the library. It mainly supports SVG rendering by utilising the underlying vector graphics in modern web browsers. It also includes the feature to render entirely using HTML5 canvas, but at the moment of writing this option limits the range of functionality available and is only suitable for simple diagrams. *JGraphX* is primarily designed to be used in a desktop environment. It enables developers to produce Java Swing appellations that feature interactive diagramming functionality.

Example applications for *mxGraph* include process diagrams, workflows, flowcharts and network visualisations. One recent implementation of *mxGraph* in a bioinformatics project is the development of a knowledge-based decision support system to study protein complexes [98].

### 4.6 JUNG

The *Java Universal Network/Graph (JUNG)* framework [99] (http://jung.sourceforge.net/) is an open source library that provides a common and extendible language for the modelling, analysis and visualisation of data using graphs or networks. *JUNG's* architecture is designed to support a variety of representations of entities and their relationships: directed and undirected graphs, multi-modal graphs, graphs with parallel edges and hypergraphs. Also, it provides a mechanism for annotating graphs, nodes and links with metadata.

By July 2014, the distribution of *JUNG* (version 2.0) includes the implementation of a number of algorithms from graph theory, data mining and social network analysis. Some examples are routines for clustering, decomposition, optimisation, random graph generation, statistical analysis and calculation of network distances, flows and other measurements (e.g. centrality, PageRank and HITS). Furthermore, *JUNG* also provides a visualisation framework that makes easy the construction of tools for the interactive exploration of network data.

In the proteomics field, the *JUNG*'s graph algorithms have been used by the *mzJava* library (http://mzjava.expasy.org/), for the analysis of MS data from large-scale proteomics and glycomics experiments. In other fields, one example is GBOOST [100], a GPU (Graphics Processing Unit)-based tool for detecting gene–gene interactions in genome–wide case control studies, which makes use of *JUNG* to visualise the resulting graphs.

### 4.7 Other libraries

Several other JavaScript-based libraries are also available for representing networks. For instance, *Arbor.js* (http://arborjs.org/) is a lightweight graph visualisation library based on jQuery. It provides force-directed layout plus graph data structure and screen refresh handling. *Arbor.js* is not restricted to a specific screen drawing method. This means that developers are free to choose among HTML5 canvas, SVG or event positioned HTML elements.

*VivaGraphJS* (https://github.com/anvaka/VivaGraphJS) is another free to use graph-drawing library based on JavaScript that supports force-directed layout. Different rendering engines are also supported including WebGL, SVG or CSS formats. Compared to other alternatives, *VivaGraphJS* can provide a better performance and works well with medium size networks (of around 5000 nodes).

For dynamic networks, *GraphStream* [101] (http://graphstream-project.org/) is a Java-based library whose main purpose is to address the existing challenges in modelling entity relationships in an evolving environment. It can handle the evolution of graphs, which can be described as the changes on the values stored on the edges and nodes of a graph in time. Its design relies on an event-based engine allowing several event sources. Events can then be generated from other parts of the application using *GraphStream*, read from a file or received from a remote server.

### 4.8 Remarks

Although node-link diagrams constitute the most familiar representation of graphs, there are alternative ways of representing this type of information. Node-link diagrams are good for showing the overall structure of a sparse graph but they can become unreadable in dense networks. In contrast, adjacency matrix representations are particularly effective for dense graphs (Supporting Information Fig. 2) and can be used to highlight various attributes of nodes and their links. The main drawback of this approach is that it does not show the topology of the network and is ill-suited for the representation of pathways [70].

There have been recent attempts to create innovative ways of visualising networks. For instance, NodeTrix [102,103] tried to combine node-link charts with adjacency matrix diagrams, to take advantage of both worlds (Fig.3B). Hive plot [104] is a new way of generating node-link diagrams (Fig.3C). It defines a linear layout for nodes, grouping and arranging nodes based on their attributes. The goal is to utilise human's positional visual channel more effectively. Another example is PivotPaths [105], which tried to address the need of a filtering functionality in complex networks by introducing faceting (Supporting Information Fig. 3). It exposes faceted relationships as visual paths and this arrangement enables developers to navigate in a better way throughout the information space.

It is also worth mentioning here the concept of an Arc diagram [106], which is a style of graph drawing in which the vertices of a graph are placed along a line in the Euclidean plane with edges being drawn as semicircles in one of the two half planes bounded by the line or as smooth curves formed by sequences of semicircles (Fig.3D). Heer et al. [56] recommended that these representations may not convey the overall structure of the graph as effectively as a 2D representation, but that their layout made easy the display of multivariate data associated with the vertices of the graph.

The underlying data structure to represent networks and graphs can greatly impact the efficiency and effectiveness of the front-end visualisation. In biology, it has been observed a rapid increase in data volumes and complexity. In parallel, there is the need to integrate more and more heterogeneous data sets. Therefore, the traditional way of storing every node and link in memory or to use a relational database to do this is often no longer feasible and more scalable solutions are needed. In this context, high hopes have been deposited in the development of graph databases such as Neo4j (http://www.neo4j.org/), FlockDB (https://github.com/twitter/flockdb) and AllegroGraph (http://franz.com/agraph/allegrograph/). All of them use graph structures with nodes, edges and attributes to represent and store data. Compared with the traditional relational databases, graph databases can scale more naturally to large graph data sets. They are particularly good at handling graph-type queries like for example, computing the shortest path between two nodes. As a result, there is an increasing adoption of graph databases as the backend for implementing network visualisations. For instance, *Gephi* already provides a plugin to connect to a Neo4j database (https://marketplace.gephi.org/plugin/neo4j-graph-database-support/) and another project not mentioned before called Linkurious (http://linkurio.us/) provides a web-based platform to explore and visualise networks stored in Neo4j databases.

## 5 Hierarchies

Visualising hierarchical data has its own objectives and challenges [107]. Common reasons to use hierarchies are, among others, to gain insight on the structure of a hierarchy, to understand the distribution of data within the context of the structure, or to provide a summary of the data set into aggregated data, in order to avoid information overload.

There is a selection of visualisation techniques that can be used in hierarchies. They generally fall into two categories: (i) Node-link diagrams such as trees, dendrograms, radial trees and hyperbolic trees. They are focused on highlighting the relationships between data items using visible graphical edges between the parent and child nodes and (ii) Space-filling diagrams such as treemaps, where the emphasis is put on the relative sizes of the nodes within the hierarchy where areas are recursively subdivided into rectangles. The adjacency diagrams constitute another example within this category, where nodes are drawn as solid areas in either arcs or rectangles. The size of the nodes encodes its quantitative attribute and their relative position reveals their position in the hierarchy. This way, the size of any node in the tree is quickly revealed, which offers a better readability and a size estimation. In addition, enclosure diagrams use the concept of containment to represent the hierarchy, since the size of each leaf node's circle reveals the quantitative dimension of each data point and the enclosing circles show the approximate cumulative size of each sub-tree.

Much of the biological data has a natural hierarchy and many research projects focus on extracting these representations. Phylogenetic trees [108] represent one of the best examples by showing the inferred evolutionary relationships among various taxonomical entities. Another example is the application of hierarchies to help solving the protein inference in proteomics [109], where dendrograms can be used for interpreting the results.

In this section, we will cover four libraries that support hierarchical data visualisations (Table1). A list of the most common hierarchical layouts together with a short description of each one can be found in the Supporting Information Table 3.

### 5.1 D3.js

**Figure 4:**
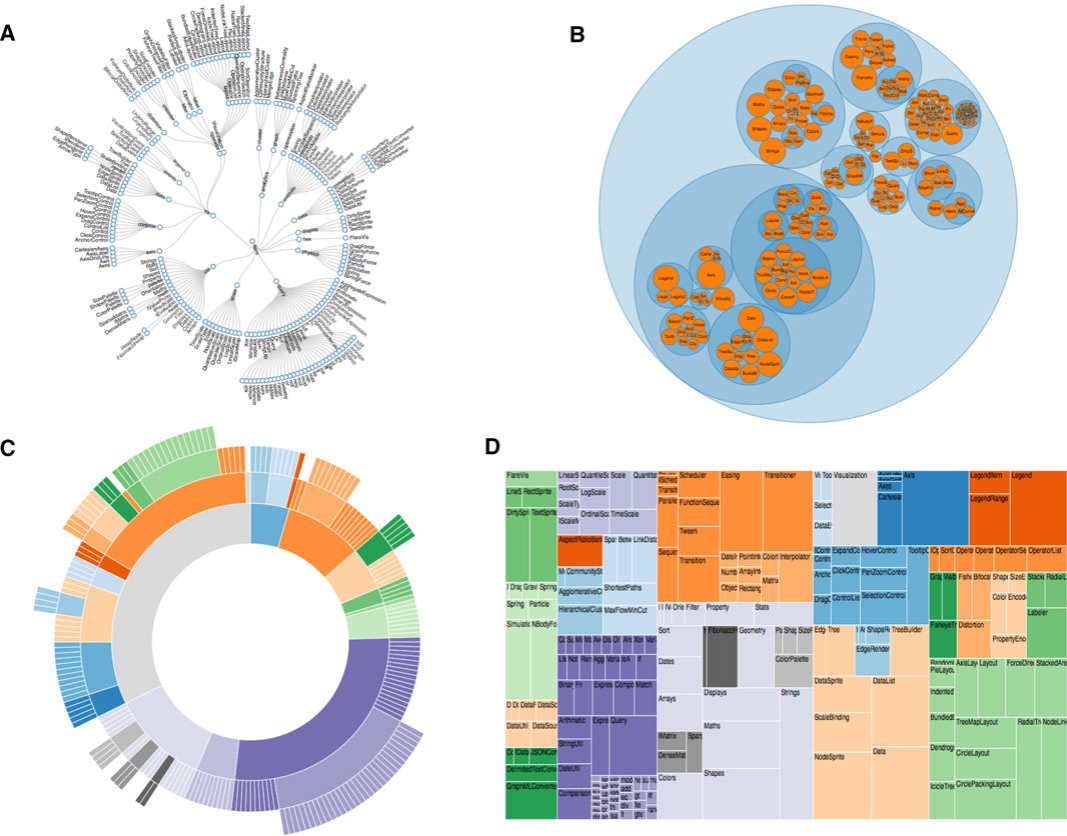
Examples of visualisation for hierarchical data: (A) Tree radial layout, (B) Pack layout, (C) Sunburst partition layout, and (D) Treemap layout. All the examples were generated using the *D3.js* library.

The *D3.js* library can treat hierarchical data visualisation as a layout. In fact, its API implements a number of frequently used layouts for different types of data, including a group of them for hierarchical data. These layouts follow the same basic structure: the input to the layout is the root node of the hierarchy and the output is an array representing the computed positions of all the nodes. For the node-link diagram approach, the classic tree layout produces node-link diagrams using the Reingold-Tilford ‘tidy’ algorithm [110] (Fig.4A and Supporting Information Fig. 4) and the cluster layout produces dendrograms, which cluster and place leaf nodes of the tree at the same depth.

In the case of the space-filling approach, the pack layout produces enclosure diagrams (Fig.4B). In addition, the partition layout produces adjacency diagrams, providing two orientations: the Cartesian orientation is often called ‘Icicle tree’ (Supporting Information Fig. 5), whereas the radial orientation is called ‘sunburst’ (Fig.4C). Finally, the treemap layout produces treemaps (Fig.4D). The layout algorithms for the treemaps can be customized: squarify for rectangular subdivision, slice for horizontal subdivision, dice for vertical subdivision and slice-dice for alternating between horizontal and vertical subdivisions. These layouts produced by the *D3.js* library have been applied to explore large biological data sets. For instance, VisXplore [111] is a visualisation system for analysing complex clinical data sets which can use both the cluster and the treemap layouts.

### 5.2 Google charts

*Google Charts* provides two types of charts for hierarchical data: Org charts and treemap charts. Org charts are node-link diagrams that are designed to show relationships in a given organisation. However, they can also be used to show tree structures such as family trees. The expected input data format for this chart type is a table containing three string columns. The first two columns are used for describing parent-child relationships whereas the last column is used for showing text as tooltips. The treemaps also expect input data in a predefined tabular format, containing one additional column that is used to represent the size of the node.

### 5.3 matplotlib

As a plotting library, *matplotlib* focuses on the fundamental components needed for constructing high-quality charts. However, it does not provide charting capabilities for hierarchical data ‘out-of-the-box’. Instead, developers can take advantage of other Python packages in its computing ecosystem. For node-link diagrams, *matplotlib* can be used together with *networkx* [112] (https://networkx.github.io/), a Python package for creating and manipulating complex network data structures, for example, circular trees (http://networkx.lanl.gov/examples/drawing/circular_tree.html).

However, its power lies in the possible integration with other Python-based software packages used in mathematics, science and engineering such as *NumPy* [113] (http://www.numpy.org/) and *SciPy* [114] (http://www.scipy.org/). *NumPy* provides support for mathematical data structures such as arrays and matrices, along with a collection of other high-level mathematical functions. *SciPy* contains additional routines, for example, for solving differential equations and sparse matrices. Combining *matplotlib* with *SciPy*, which also contains functions for hierarchical clustering, can generate dendrograms. Space-filling diagrams such as treemaps can also be generated using *matplotlib* and *SciPy* together (http://wiki.scipy.org/Cookbook/Matplotlib/TreeMap).

### 5.4 Graphviz

*Graphviz* supports various layouts for hierarchical data. They come as a collection of programs that can be run on the command line. The expected input is composed by hierarchical data described in the DOT language and the output can be exported to different formats (e.g. PostScript or SVG). For the node-link diagram approach, it supports the standard tree layout and the force directed layout. Also, it provides radial layouts, where the root node is placed at the centre, whereas the remaining nodes are placed on a sequence of concentric circles centred on the root node. In the case of the space-filling diagram approach, *Graphviz* can draw the tree as a squarified treemap [115] or as a packed cluster structure using the pack layout.

*Graphviz* has been already used in bioinformatics. One example is SCOP2 [116], a project focused on the visualisation of the SCOPs. In particular, SCOP2-graph (http://scop2.mrc-lmb.cam.ac.uk/graph/index.html) is a web-based viewer for the display and navigation through the classifications using *Graphviz*. Other example is OrthoMaM [117], which is a database of orthologous exons and coding sequences in mammals used for comparative genomics. It uses *Graphviz* for visualising the details of the GO annotations.

### 5.5 Other libraries

As hierarchies are considered to be a subset of general graphs, the majority of the libraries mentioned in the networks section are capable of displaying hierarchical data as trees. For instance, *Cytoscape* supports both the tree and the radial layouts, whereas *mxGraph* supports a range of hierarchical layouts.

### 5.6 Remarks

The choice of the layout is critical for the visualisation of hierarchical data. Node-link diagrams are great for showing the structure of a hierarchy. However, the display is not compact and the required space increases polynomially as the number of nodes increases. Space-filling diagrams are effective representations of two attributes beyond node-link diagrams by using colour and area, but they are not as good at representing structures.

There are alternative layouts to overcome these shortcomings. One popular technique is to display trees in 3D instead of 2D. The goal is then to utilise the extra dimension to help easing the problem of displaying large structures. The cone tree [118] is one of the best known 3D tree layout techniques. Another technique is the hyperbolic layout [119], which employs a hyperbolic space that has more room than the Euclidean space, resembling the effect of using fish-eye lenses on traditional tree layouts. This distorted view makes possible the interaction with large trees.

## 6 Future challenges

The on-going explosion of biological data presents a significant challenge to existing visualisation libraries. Since most of the libraries focus on handling data sets of small or intermediate size, the majority of them are not designed having scalability in mind. The advance of parallel computing and GPU-based computing allows visualisation libraries to tap into the raw computing power of the hardware. However, this approach often requires domain specific knowledge to understand the platform or the parallelisation mechanism. To overcome these problems, there are already attempts to make this process more transparent to developers. For instance, Superconductor (http://superconductor.github.io/superconductor/) provides a web framework that automatically compiles and parallelizes algorithms to support visualisation of hundreds and thousands of data points.

Additionally, most of the existing visualisation libraries adopted a ‘Swiss-army knife’ approach, since they tried to achieve broader adoption by offering a large collection of visualisation solutions by default. For researchers and developers, the challenge remains on how to pick or create the most suitable visualisations for each particular task. Research teams in academic environments often do not have the in-house expertise needed. It is therefore paramount to establish a set of principles and guidelines on data visualisation. A lot of research has already been performed in systematically categorising the visualisation background and principles [120], which are widely accepted and applied in other scientific disciplines. However, many of them have not been introduced to biology to the same extent. There have been attempts on narrowing the existing gap [121] but more needs to be done.

Furthermore, visualisation libraries are greatly influenced by the underlying technology. Recently, great advances in web development techniques have taken place. This has led to a shift from traditional plugin-based frameworks such as Adobe® Flash (http://www.adobe.com/) and Silverlight® (http://www.microsoft.com/silverlight/) to more browser native approaches such as HTML5/CSS/JavaScript. While these technical advances are useful and can help to improve data visualisation, they can also become a source of confusion when choosing the library. To make things worse, the reincarnations/the new versions of the libraries can sometime offer completely different APIs containing only a subset of the original features. This requires tool developers to have in-depth knowledge of the underlying technology to make an informed judgement.

Definitely, more could be done in the context of community support and classification of these libraries. In this context, we want to highlight the VIZBI initiative (http://vizbi.org/), which brings together researchers developing and using computational visualisation tools in a broad range of biological research areas. It has proven to be the ideal environment to discuss new visualisation techniques and approaches in the context of bioinformatics and life sciences [6,122].

## 7 Conclusion

Visualisation of biological data is rapidly evolving. Its ultimate goal is to generate insights into the processes of the living organisms. To fulfil this ambition, further development of visualisation tools and techniques is needed. Open source and free-to-use visualisation libraries and frameworks play an important role in the development of new tools and the illustration of new research discoveries. They offer the right balance between innovation and reusability. They can also streamline the implementation of the common features needed in the visualisation tools and free up the developers’ time to focus on developing novel solutions.

## Abbreviations

API: application programming interface
AWT: abstract window toolkit
CATH: class, architecture, topology, homology (classification)
CSS, CSS3: cascading style sheets
DOT: graph description language
EBI: European Bioinformatics Institute
ENA: European Nucleotide Archive
GO: Gene Ontology
GPU: Graphics Processing Unit
GRAL: GRAphing Library
GUI: graphical user interface
HTML, HTML5: hyper text markup language
JSON: javascript object notation
JSP: java server pages
JUNG: java universal network/graph (framework)
PIES: protein interaction extraction system
SCOP: Structural Classification Of Proteins
SNP: Single Nucleotide Polymorphism
SVG: scalable vector graphics
SWT: standard widget toolkit
UI: user interface
URI: uniform resource identifier
VML: vector markup language
WebGL: Web Graphics Library
