# Supplementary material for: Open source libraries and frameworks for biological data visualisation: A guide for developers
Source: Proteomics. 2015 Feb 5;15(8):1356–74. doi: 10.1002/pmic.201400377 (PMC4409855; doi:10.1002/pmic.201400377)
Supplement: Supplementary file 1 [file pmic0015-1356-sd1.docx]

**Supplementary Tables**

**Supplementary Table 1.** List of the different types of plots and charts supported by the different libraries described in the main text.

| **Chart Type** | **Description** | **JFreeChart** | **Google Charts** | **D3.js** | **matplotlib** | **GRAL** | **Jzy3d** | **XChart** | **Flot** | **Bokeh** | **Highcharts** |
| --- | --- | --- | --- | --- | --- | --- | --- | --- | --- | --- | --- |
| Bar Chart | A **bar graph** is a chart that uses either horizontal or vertical bars to show comparisons among categories. One axis of the chart shows the specific categories being compared, and the other axis represents a **discrete** value | ✓ | ✓ | ✓ | ✓ | ✓ | ✓ | ✓ | ✓ | ✓ | ✓ |
| Bar Chart with negative values | A **bar graph** chart that allows negative values | ✗ | ✗ | ✓ | ✗ | ✗ | ✗ | ✗ | ✗ | ✗ | ✓ |
| Stacked Bar Chart | The **stacked bar chart** is a bar chart that represents different groups on top of each other. The height of the resulting bar shows the combined result of the groups | ✓ | ✓ | ✓ | ✓ | ✗ | ✗ | ✗ | ✓ | ✓ | ✓ |
| Box Chart | In descriptive statistics, a **box plot** or **boxplot** is a convenient way of graphically depicting groups of numerical data through their quartiles | ✓ | ✓ | ✓ | ✓ | ✓ | ✗ | ✗ | ✓ | ✓ | ✗ |
| Bubble Chart | A **bubble chart** is a type of chart that displays three dimensions of data. Each entity with its triplet (*v*_1_, *v*_2_, *v*_3_) of associated data is plotted as a circle. The *v_i_* and *v*_2_ values are represented by the *xy* axis and *v*_3_ through the size of the circle. | ✓ | ✓ | ✓ | ✓ | ✓ | ✗ | ✗ | ✗ | ✓ | ✓ |
| Histogram | In statistics, a **histogram** is a graphical representation of the distribution of data. It is an estimate of the probability distribution of a continuous variable. The total area of the histogram is equal to the number of data points. | ✓ | d  Example✓ | ✓ | ✓ | ✓ | d  Example✓ | ✓ | ✓ | ✓ | ✓ |
| Pie Chart | A **pie chart** is a circular chart divided into sectors, illustrating numerical proportion. In a pie chart, the arc length of each sector (and consequently its central angle and area) is proportional to the quantity it represents | ✓ | d  Example✓ | ✓ | ✓ | ✓ | d  Example✓ | ✗ | ✓ | ✓ | ✓ |
| Semi Circle Donut | A half customised pie plot that leaves a hole in the middle | ✗ | ✓ | ✓ | ✗ | ✗ | ✗ | ✗ | ✗ | ✗ | ✓ |
| Scatter Plot | A **scatter plot**, **scatterplot** or **scattergraph** is a type of mathematical diagram using Cartesian coordinates to display values for two variables for a set of data. The data is displayed as a collection of points, each having the value of one variable determining the position on the horizontal axis and the value of the other variable determining the position on the vertical axis | ✓ | d  Example✓ | ✓ | ✓ | ✓ | d  Example✓ | ✓ | ✓ | ✓ | ✓ |
| Area Chart | An **area chart** or **area graph** displays graphically quantitative data. It is based on the line chart. The area between axis and line are commonly emphasized with colours, textures and hatchings. The Area charts are used to represent cumulated totals using numbers or percentages (**stacked area charts** in this case) over time | ✓ | d  Example✓ | ✓ | ✓ | ✓ | d  Example✓ | ✓ | ✓ | ✓ | ✓ |
| Scatter Matrix | In multivariate statistics and probability theory, the scatter matrix is a type of statistics that is used to make estimates of the covariance matrix of the multivariate normal distribution | ✗ | ✗ | ✓ | ✓ | ✗ | ✗ | ✗ | ✗ | ✗ | ✗ |
| Radar Chart | A radar chart is a graphical method of displaying multivariate data in the form of a two-dimensional chart of three or more quantitative variables represented on axes starting from the same point | ✗ | ✗ | ✓ | ✓ | ✗ | ✗ | ✗ | ✗ | ✗ | ✓ |

**Supplementary Table 2.** List of the different types of network layouts supported by the different libraries described in the main text.

| **Layout** | **Description** | **Cytoscape** | **Gephi** | **GraphViz** | **Sigma.js** | **mxGraph** | **JUNG** |
| --- | --- | --- | --- | --- | --- | --- | --- |
| Force Directed | A **force directed** layout is a family of layouts that position the nodes of a graph by assigning forces among the set of edges and the set of nodes, based on their relative positions, and then make use of these forces to either simulate the motion of the edges and nodes, or to minimize their energy | ✓ | ✓ | ✓ | ✓ | ✓ | ✓ |
| Spring | A **spring** layout is a one method of the **force directed** layout family | ✓ | ✗ | ✓ | ✗ | ✓ | ✓ |
| Circular | A **circular** layout is a style of graph drawing that places the nodes on a circle. The nodes are often evenly spaced | ✓ | ✓ | ✓ | ✗ | ✗ | ✓ |
| Stack | A **stack** layout takes a two-dimensional array of data and computes a baseline. The baseline is then propagated to the above layers | ✓ | ✗ | ✗ | ✗ | ✓ | ✗ |
| Grid | A **grid** layout draws a network in a 2D grid where each node is placed in its own cell so that edge intersections will be minimized | ✓ | ✗ | ✗ | ✗ | ✗ | ✗ |
| Orthogonal | A **orthogonal** layout places all the edges of the network to run horizontally or vertically, parallel to the coordinate axes of the layout | ✓ | ✗ | ✗ | ✗ | ✗ | ✗ |
| Self-organising Map (SOM) | A **self-organising map** layout is a drawing technique that reduces the dimensions of the data through the use of self-organising neural networks | ✓ | ✗ | ✗ | ✗ | ✗ | ✓ |
| Yifan Hu | A **Yifan Hu** layout algorithm is both efficient and suitable for representing large networks. It combines a multilevel approach that effectively overcomes local minimums. Also, it uses an adaptive cooling scheme for selecting the optimal depth of octree/quadtree | ✗ | ✓ | ✗ | ✗ | ✗ | ✗ |
| Force Atlas | A **force atlas** layout is one type of **force directed** layout. It runs continuously as long as the nodes repulse and the edges attract. It also changes the forces or how they are simulated during the execution | ✗ | ✓ | ✗ | ✗ | ✗ | ✗ |
| Composite | The **composite** layouts combine the different types of layouts in drawing networks | ✗ | ✗ | ✗ | ✗ | ✓ | ✓ |
| Parallel Edge | A **parallel edge** layout is a layout algorithm that routes parallel edges (edges that connect the same pair of nodes) of a graph | ✗ | ✗ | ✗ | ✗ | ✓ | ✗ |
| Static | The **Static** layout place nodes in the locations given by the user | ✗ | ✗ | ✗ | ✗ | ✗ | ✓ |

.

**Supplementary Table 3.** List of the different types of hierarchical layouts supported by the different libraries described in the main text.

| **Layout** | **Description** | **D3** | **Google Charts** | **Matplotlib** | **GraphViz** | **Cytoscape** | **Gephi** | **mxGraph** | **JUNG** |
| --- | --- | --- | --- | --- | --- | --- | --- | --- | --- |
| Tree | The **tree** layout produces tidy node-link diagrams of trees | ✓  Example: <http://tinyurl.com/jwccd2z> | ✓  Example: <http://tinyurl.com/m9zxgw9> | ✓ | ✓ | ✓ | ✗ | ✗ | ✗ |
| TreeMap | A **treemap** recursively subdivides the area into rectangles. Similar to adjacency diagrams, the size of any node in the tree is quickly revealed | ✓  Example: <http://tinyurl.com/n7pjgfy> | ✓  Example: <http://tinyurl.com/p6kyzem> | ✓ | ✓ | ✗ | ✗ | ✗ | ✗ |
| Dendrogram | A **dendrogram** is a node-link diagram that places leaf nodes of the tree at the same depth. It is frequently used to illustrate the arrangement of the clusters produced by hierarchical clustering. | ✓  Example: <http://tinyurl.com/ml9veer> | ✗ | ✓ | ✗ | ✗ | ✗ | ✗ | ✗ |
| Pack | A **pack** layout produces enclosure diagrams using containment to represent the hierarchy. The size of each leaf node’s circle reveals a quantitative dimension of each data point. The enclosing circles show the approximate cumulative size of each sub-tree | ✓  Example: <http://tinyurl.com/njgqeao> | ✗ | ✗ | ✓ | ✗ | ✗ | ✗ | ✗ |
| Radial | A **radial tree** or **radial map** is a method of drawing a tree structure that expands radially. The layout is generated by working outwards from the centre root node | ✓ | ✗ | ✗ | ✓ | ✗ | ✓ | ✗ | ✓ |
| Partition | The **partition** layout produces adjacency diagrams. It is a space-filling variant of a node-link tree diagram. Rather than drawing a link between nodes, nodes are drawn as solid areas. Their placement relative to other nodes reveals their position in the hierarchy | ✓  Example: <http://tinyurl.com/kebwho2> | ✗ | ✗ | ✗ | ✗ | ✗ | ✓ | ✗ |
| Balloon | A **balloon** layout assigns positions to tree nodes using associations with nested circles (“balloons”). A balloon is nested inside another balloon if the first balloon’s sub-tree is a sub-tree of the second balloon’s sub-tree | ✓ | ✗ | ✗ | ✗ | ✗ | ✗ | ✗ | ✓ |

.

**Supplementary Figures**


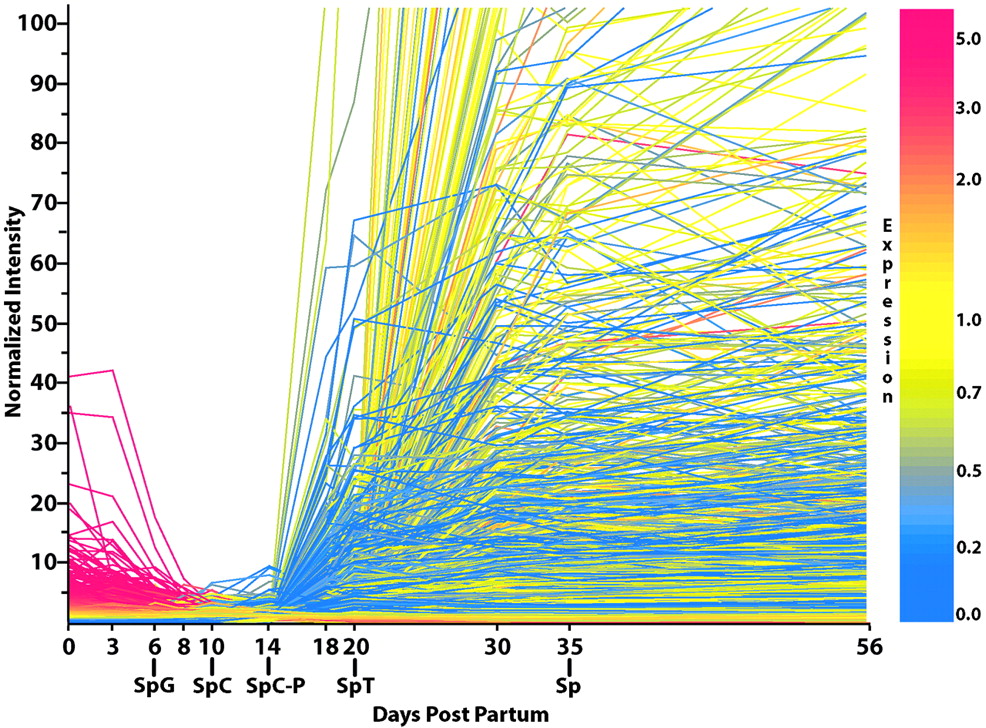


**Supplementary Figure 1**. Example of chart: Parallel-coordinate plot of statistically significant transcripts during testis development (original manuscript published by Shima J. E. *et al.* *Biol Reprod* 2004;71:319-330).


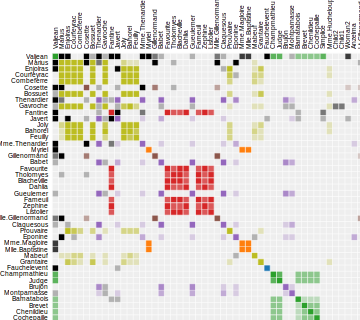


**Supplementary Figure 2**. Adjacency matrix representation generated using *D3.js* (<http://bost.ocks.org/mike/miserables/>)*.*

*
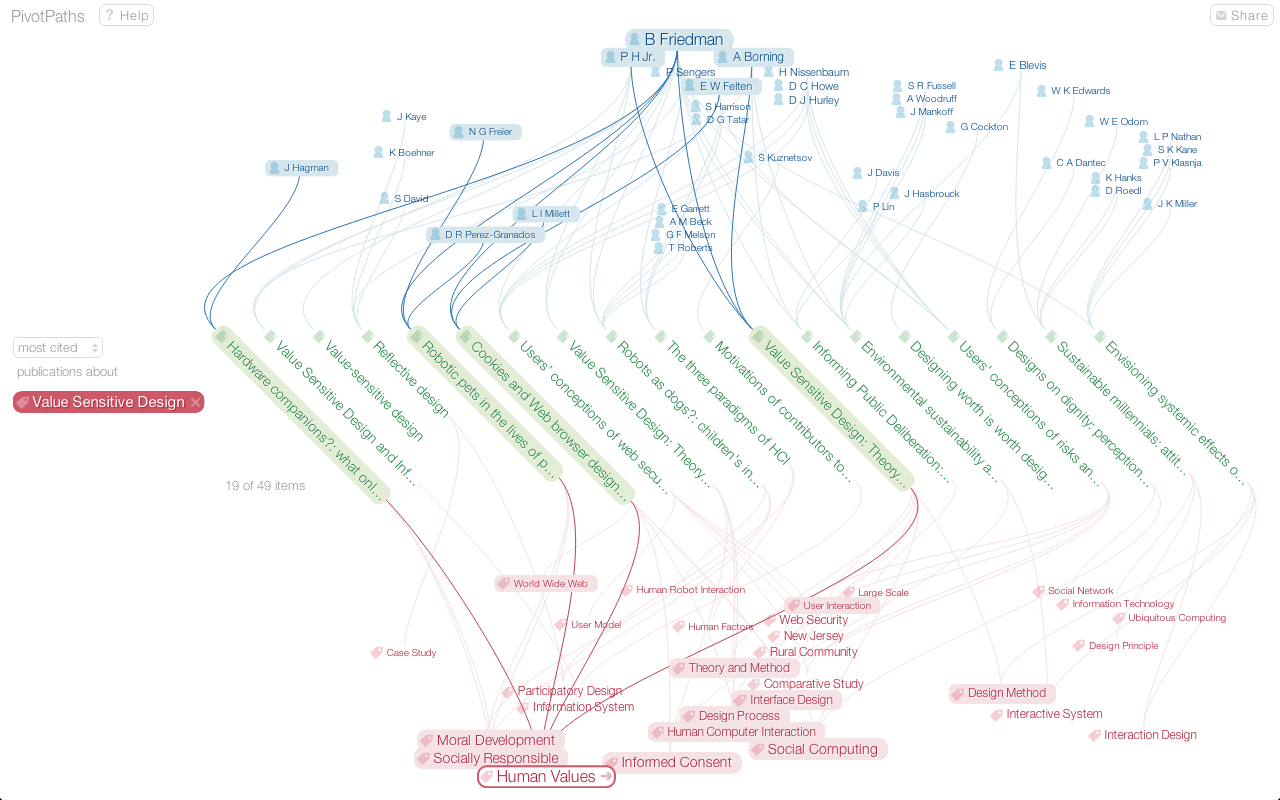
*

**Supplementary Figure 3.** Example of network: *PivotPaths* visualisation for exploring faceted information (<http://mariandoerk.de/pivotpaths/>).


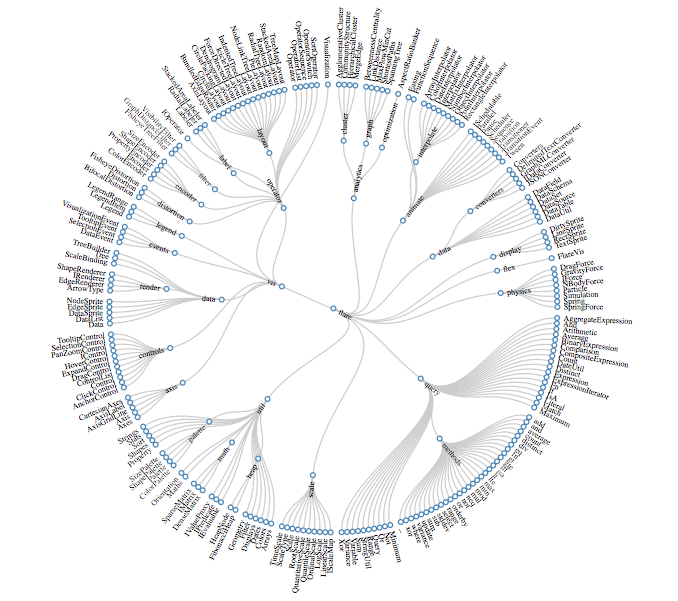


**Supplementary Figure 4.** Example of visualisation for hierarchical data: Circular layout, generated using the *D3.js* library.


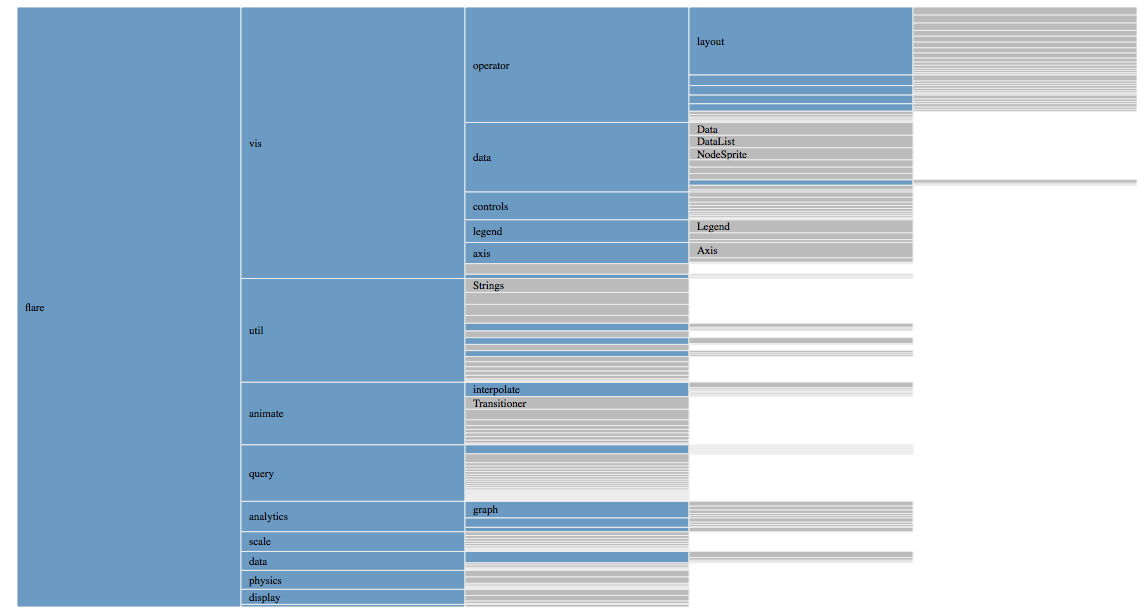


**Supplementary Figure 5.** Example of visualisation for hierarchical data: Icicle partition layout, generated using *D3.js* library.
